# Primacy coding facilitates effective odor discrimination when receptor sensitivities are tuned

**DOI:** 10.1371/journal.pcbi.1007188

**Published:** 2019-07-19

**Authors:** David Zwicker

**Affiliations:** 1 Max Planck Institute for Dynamics and Self-Organization, Göttingen, Germany; 2 John A. Paulson School of Engineering and Applied Sciences, Harvard University, Cambridge, Massachusetts, United States of America; 3 Kavli Institute for Bionano Science and Technology, Harvard University, Cambridge, Massachusetts, United States of America; UCL, UNITED KINGDOM

## Abstract

The olfactory system faces the difficult task of identifying an enormous variety of odors independent of their intensity. Primacy coding, where the odor identity is encoded by the receptor types that respond earliest, might provide a compact and informative representation that can be interpreted efficiently by the brain. In this paper, we analyze the information transmitted by a simple model of primacy coding using numerical simulations and statistical descriptions. We show that the encoded information depends strongly on the number of receptor types included in the primacy representation, but only weakly on the size of the receptor repertoire. The representation is independent of the odor intensity and the transmitted information is useful to perform typical olfactory tasks with close to experimentally measured performance. Interestingly, we find situations in which a smaller receptor repertoire is advantageous for discriminating odors. The model also suggests that overly sensitive receptor types could dominate the entire response and make the whole array useless, which allows us to predict how receptor arrays need to adapt to stay useful during environmental changes. Taken together, we show that primacy coding is more useful than simple binary and normalized coding, essentially because the sparsity of the odor representations is independent of the odor statistics, in contrast to the alternatives. Primacy coding thus provides an efficient odor representation that is independent of the odor intensity and might thus help to identify odors in the olfactory cortex.

## Author summary

Humans can identify odors independent of their intensity. Experimental data suggest that this is accomplished by representing the odor identity by the earliest responding receptor types. Using theoretical modeling, we here show that such a primacy code outperforms alternative encodings and allows discriminating odors with close to experimentally measured performance. This performance depends strongly on the number of receptors considered in the primacy code, but the receptor repertoire size is less important. The model also suggests a strong evolutionary pressure on the receptor sensitivities, which could explain observed receptor copy number adaptations. By predicting psycho-physical experiments, the model will thus contribute to our understanding of the olfactory system.

## Introduction

The olfactory system identifies and discriminates odors for solving vital tasks like navigating the environment, identifying food, and engaging in social interactions. These tasks are complicated by the enormous variety of odors, which vary in composition and in the concentrations of their individual molecules. In particular, the olfactory system needs to separately recognize the odor identity (what is there?) and the odor intensity (how much is there?). For instance, the identity is required to decide whether to approach or avoid an odor source, whereas the intensity information is important for localizing it. It is not understood how these two odor properties are separated and how odors are discriminated reliably.

Odors are comprised of chemicals that bind to and excite olfactory receptors in the nose in mammals and on antenna in insects. Each receptor responds to a wide range of odors and each odor activates many receptor types. The resulting combinatorial code allows to distinguish odor identities [1–3], but also depends on the odor intensity, since receptors respond stronger to more concentrated molecules [4]. To separate these two properties, the neural signals are processed in the olfactory bulb (antennal lobe in insects) and then forwarded to the olfactory cortex, where odors are identified by comparing to memorized patterns. Indeed, experiments indicate that the olfactory cortex receives a concentration-invariant code [5, 6], which allows to identify odors irrespective of their intensity. Consequently, the olfactory bulb can be thought of as a signal processor that removes statistical redundancies in the input to provide a more useful representation to the olfactory cortex. However, so far it is not clear what processing the olfactory bulb performs and how this affects odor representations.

The olfactory bulb contains neural clusters called glomeruli, which each receive input from a specific receptor type [7–9]. Each glomerulus excites associated projection neurons, which project into the olfactory cortex. Additionally, the glomeruli are connected by local neurons [10, 11], which inhibit the projection neurons [12–18]. These local neurons could mediate a global normalization resulting in an intensity-invariant representation of the odor identity [19, 20]. However, we showed that simple normalized representations still depend strongly on the number of ligands in a mixture and might thus not be optimal for solving olfactory tasks [21]. An alternative to these normalized representations is rank coding, where the order in which the receptors are excited is used to encode the odor identity robustly and independently of the odor intensity [22]. Indeed, experiments suggests that odors are encoded robustly by the receptor types that respond within a given time window after sniff onset [23–25]. In particular, the odor identity could be robustly encoded by a fixed number of the receptors that respond first, which is known as primacy coding [24, 26]. So far, it is unclear how efficient and useful primacy coding is and how it compares to alternative schemes.

In this paper, we consider a simple model of primacy coding and investigate how well it represents complex odors. In particular, we identify how much information is transmitted to the cortex and how well this information can be used to perform typical olfactory tasks, like identifying the addition of a target odor to a background or discriminating odor mixtures. Our statistical approach allows linking parameters of the primacy code to results from typical psychophysical experiments. We show that primacy coding provides a robust and compact representation of the odor identity over a wide range of odors, independent of the odor intensity, and that it outperforms other simple coding schemes. However, this good performance of the olfactory system hinges on tuned receptor sensitivities, which suggests that there is a strong selective pressure to adjust the sensitivities on evolutionary and shorter timescales.

## Results

We describe odors by concentration vectors $c=(c_{1},c_{2},\ldots ,c_{N_{L}})$, which determine the concentrations *c*_*i*_ ≥ 0 of all ligands that can be detected by the olfactory receptors. The number *N*_L_ of possible ligands is at least *N*_L_ = 2300 [27] although the actual number is likely much larger [28]. Typical odors contain only tens to hundreds of ligands, implying that most *c*_*i*_ are zero.

In experiments, the olfactory system is typically characterized by presenting odors with particular statistics, e.g., by choosing mixtures from a given ligand library. Although such experiments allow to characterize the olfactory system in a part of odor space, we ultimately want to understand how the system performs in its natural environment. Unfortunately, the statistics of natural odors are difficult to measure [29], so we here consider a broad class of odor distributions to approximate natural odor statistics [30]. In particular, we consider a situation in which each ligand *i* has a probability *p*_*i*_ to appear in an odor. For simplicity, we neglect correlations in their appearance, so the mean number *s* of ligands in an odor is *s* = ∑_*i*_ *p*_*i*_. To model the broad distribution of ligand concentrations, we choose the concentration *c*_*i*_ of ligand *i* from a log-normal distribution with mean *μ*_*i*_ and standard deviation *σ*_*i*_ if the ligand is present. Consequently, the mean concentration of a ligand in any odor reads 〈*c*_*i*_〉 = *p*_*i*_ *μ*_*i*_ and the associated variance is $\mathrm{var}\left(c_{i}\right)=\left(p_{i}-p_{i}^{2}\right)\mu_{i}^{2}+p_{i}\sigma_{i}^{2}$. For simplicity, we consider ligands with equal statistics in this paper, so the distribution *P*_env_(***c***) of odors is characterized by the three parameters *p*_*i*_ = *p*, *μ*_*i*_ = *μ*, and *σ*_*i*_ = *σ*. Using these broad odor statistics and more specific ones will allow us to analyze the performance of olfactory models in natural environments and in typical psycho-physical experiments, respectively.

### Simple model of primacy coding

**Fig 1:**
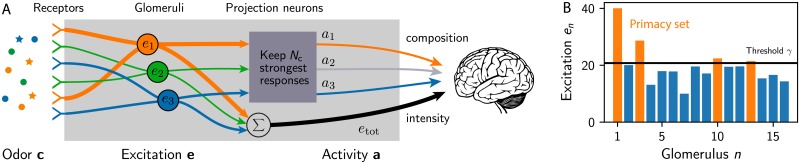
Simple model of primacy coding. (A) Schematic picture of the signal processing in the olfactory bulb: An odor comprised of many ligands excites the olfactory receptors and the signals from all receptors of the same type are accumulated in respective glomeruli. Under primacy coding, the glomeruli with the strongest (earliest) excitations encode the odor composition, whereas the odor intensity could be encoded separately. (B) Excitations of *N*_R_ = 16 glomeruli for an arbitrary odor. The *N*_C_ = 4 glomeruli with the highest excitations, above the threshold *γ*, form the primacy set (orange bars).

Odors are detected by an array of receptors in the nasal cavity in mammals and on the antenna in insects. The receptor array consists of *N*_R_ different receptor types, which each are expressed many times. Typical numbers are *N*_R_ ≈ 50 in flies [7], *N*_R_ ≈ 300 in humans [31], and *N*_R_ ≈ 1000 in mice [32]. The excitations of all receptors of the same type are accumulated in an associated glomerulus in the olfactory bulb in mammals and the antennal lobe in insects [33]. Since this convergence of the neural information mainly improves the signal-to-noise ratio, we here capture the excitation of the receptors at the level of glomeruli; see Fig 1A. The excitation *e*_*n*_ of glomerulus *n* can be approximated by a linear function of the odor ***c*** [4, 34],

$$\begin{array}{c} e_{n}=\sum_{i=1}^{N_{L}}S_{ni}c_{i}\;, \end{array}$$ (1)

where *S*_*ni*_ denotes the effective sensitivity of glomerulus *n* to ligand *i*. Note that *S*_*ni*_ is proportional to the copy number of receptor type *n* if the response from all individual receptors is summed [30].

The sensitivity matrix *S*_*ni*_ could in principle be determined by measuring the response of each glomerulus to each possible ligand. However, because the numbers of receptor and ligand types are large, this is challenging and only parts of the sensitivity matrix have been measured, e.g., in humans [35] and flies [36]. Using these data, we showed that the measured matrix elements are well described by a log-normal distribution with a standard deviation λ ≈ 1 of the underlying normal distribution [30]. Motivated by these observations, we here consider random sensitivity matrices, where each element *S*_*ni*_ is chosen independently from the same log-normal distribution, which is parameterized by its mean $\langle S_{ni}\rangle =\bar{S}$ and variance $\mathrm{var}\left(S_{ni}\right)={\bar{S}}^{2}\left(e^{\lambda^{2}}-1\right)$ with λ = 1 [30]. Since these receptor sensitivities are broadly distributed, they might not include specific receptors related to innate behavior [37], but they can collectively discriminate concentration differences of several orders of magnitude [30].

The odor representation on the level of glomeruli excitations *e*_*n*_ depends strongly on the odor intensity, which is quantified by the total concentration *c*_tot_ = ∑_*i*_ *c_i_*. This dependency complicates the extraction of the odor identity, which is determined by the ligands which are present and their relative concentrations. A concentration-invariant representation could be achieved by normalizing the excitations by their mean [16], which leads to an efficient neural representation on the level of projection neurons [21]. However, recent experimental data suggest an alternative encoding based on the timing of the glomeruli excitation [24]. The key idea of this primacy coding is that the set of receptor types that are excited first is independent of the total concentration *c*_tot_ and thus provides a concentration-invariant representation.

In our simple model of primacy coding, odors are encoded by the identity of the *N*_C_ glomeruli that respond first. For simplicity, we here neglect the order in which they respond, in contrast to rank coding [22], and we also consider the simple situation where ligands binding to receptors only affect the magnitude of the receptor output, but not the signaling dynamics. In this case, receptors that respond first are also the ones with the largest excitation, so that the primacy code is given by the identity of the *N*_C_ glomeruli with the largest excitation, which is known as the primacy set [38].

The primacy set can be represented by a binary activity vector $a=(a_{1},a_{2},\ldots ,a_{N_{R}})$, where *a*_*n*_ = 1 implies that glomerulus *n* belongs to the primacy set and is active, while *a*_*n*_ = 0 denotes an inactive glomerulus not belonging to the primacy set. Since the active glomeruli have the highest excitation, they can be identified using an excitation threshold *γ*; see Fig 1B. Consequently, the activities are given by

$$\begin{array}{c} a_{n}=\{\begin{array}{cc} 1 & e_{n}>\gamma \left(e\right)\\ 0 & e_{n}\leq \gamma \left(e\right)\;. \end{array} \end{array}$$ (2)

Physiologically, the activities *a*_*n*_ could be encoded by projection neurons in insects and mitral and tufted cells in mammals. These neurons receive excitatory input from one glomerulus [39] and are inhibited by a local network of granule cells [20, 33]. These granule cells basically integrate the activity of all glomeruli [40] and could inhibit the glomeruli once a threshold is reached. Taken together, this would implement primacy coding since only the glomeruli that respond earliest would be activated. For simplicity, we consider the case where the number *N_C_* of active glomeruli is fixed and does not depend on the odor ***c***. The associated constraint

$$\begin{array}{c} N_{C}=\sum_{n=1}^{N_{R}}a_{n} \end{array}$$ (3)

determines the threshold *γ*. Note that the activity pattern ***a*** is sparse since only a fraction *N*_C_/**N_R_ of all glomeruli is activated. Moreover, ***a*** is independent of $\bar{S}$ and *c*_tot_, implying concentration-invariance. This is because multiplying the concentration vector ***c*** by a factor changes the excitations *e*_*n*_ and the threshold *γ* by the same factor, so that ***a*** given by Eq (2) is unaffected. In essence, only relative excitations are relevant for our model of primacy coding.

In the binary representation given by Eq (2), each receptor type can at most contribute 1 *bit* of information to the odor representation. This worst-case scenario corresponds to large processing noise, such that intermediate excitations cannot be identified in the downstream processing. In fact, the concentration range over which receptors are sensitive is typically small compared to the expected range of odorant concentrations [41]. Consequently, receptors will be activated either very little or very strongly for natural odors, suggesting a binary picture. Moreover, there is evidence that the identity of active neurons can robustly encode odor identity and is actually used in the olfactory system [5].

To see whether primacy coding encodes odor information efficiently [42], we quantify the amount of information *I* that can be learned about the odor ***c*** by observing the binary activity pattern ***a*** with given sparsity *N*_C_/*N*_R_. Since our model is deterministic, *I* is given by the entropy

$$\begin{array}{c} I=-\sum_{a}P\left(a\right)\log_{2}P\left(a\right)\;, \end{array}$$ (4)

where the probability *P*(***a***) of observing an output ***a*** depends on the odor environment *P*_env_(***c***) as well as the properties of the olfactory system, which in our model are quantified by *N*_C_, *N*_R_, and λ. Since further processing in the downstream regions of the brain can only reduce the amount of information, Eq (4) provides an upper bound for the information that the brain receives about odors when primacy coding as described by Eqs (1)–(3) is used.

In an optimal receptor array, each output ***a*** occurs with equal probability when encountering odors distributed according to *P*_env_(***c***) [30]. In our model, only outputs with exactly *N*_C_ active receptor types are permissible. The resulting representations would be optimal if each receptor type was activated with a probability 〈*a_n_* 〉 = *N*_C_/*N*_R_ and all types were uncorrelated, cov(*a*_*n*_, *a*_*m*_) = 0 for *n* ≠ *m*. The associated information

Imax(NC,NR)=log2(NRNC)≈NC-1ln2+NClog2NRNC (5)

provides an upper bound for *I* given by Eq (4). Here, the approximation on the right hand side is obtained using Stirling’s formula for large receptor repertoires (*N*_R_ ≫ *N*_C_).

### Transmitted information depends weakly on receptor repertoire

**Fig 2:**
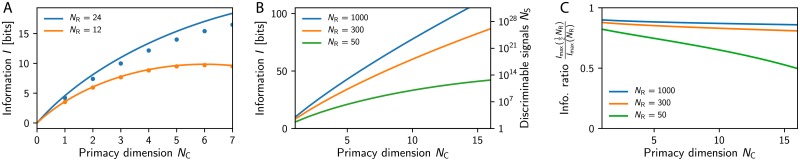
Transmitted information *I* increases strongly with primacy dimension *N*_C_ and weakly with receptor repertoire size *N*_R_. (A) The maximally transmitted information *I*_max_ (solid lines) given by Eq (5) is compared to numerical estimates of *I* (dots; *n* = 10^7^, error smaller than symbol size) obtained from ensemble averages of Eq (4). Model parameters are *N*_L_ = 512, *μ* = *σ* = 1, *s* = 16, and λ = 1, implying *ζ* ≈ 0.1. (B) *I*_max_ as a function of *N*_C_ for several *N*_R_. The right axis indicates the maximal number of distinguishable signals, *N*_S_ = 2*^I^*. (C) Reduction of *I*_max_ when half the receptor types are removed as a function of *N*_C_ for various *N*_R_.

We start by analyzing the information *I* transmitted by the primacy code using numerical ensemble averages of Eqs (1)–(4); see Methods and Models. Fig 2A shows that *I* is very close to the maximal information *I*_max_ given by Eq (5), which is obtained when all receptor types have equal activity and are uncorrelated [30]. This indicates that the primacy code uses the different receptor types with similar frequency and that correlations between them are negligible. The expression for *I*_max_ implies that the information grows linearly with the primacy dimension *N*_C_, but only logarithmically with the number *N*_R_ of receptor types. Consequently, the number of distinguishable signals, *N*_S_ = 2*^I^*, grows strongly with *N*_C_, but the dependence on the repertoire size is weaker; see Fig 2B. Given equal *N*_C_, our model thus predicts that the transmitted information in mice is only twice that of flies, although mice possess about 20 times as many receptor types. However, the number of discriminable signals changes by many orders of magnitudes, since it scales exponentially with *I*.

The logarithmic scaling of the transmitted information *I* with the receptor repertoire size *N*_R_ could explain why the ability of rats to discriminate odors is not significantly affected when half the olfactory bulb is removed in lesion experiments [43, 44]. If this operation removes half the receptor types, our model implies that the transmitted information *I* is lowered by *N*_C_ bits; see Eq (5). This corresponds to a reduction of *I* by about 10% in rats where *N*_R_ ≈ 1000; see Fig 2C. Conversely, the transmitted information decreases by almost 50% in flies, which have a much smaller receptor repertoire of *N*_R_ ≈ 50. Our model thus predicts that lesion experiments have a much more severe effect on the performance of animals with smaller receptor repertoires.

Taken together, this first analysis already suggests that the primacy code provides a robust odor representation, which is sparse, concentration-invariant, and depends only weakly on the details of the receptor array. However, for this representation to be useful to the animal, it needs to allow solving typical olfactory tasks.

### Primacy coding discriminates odors efficiently

Typical olfactory tasks include detecting a ligand in a distracting background, detecting the addition of a ligand to a mixture, as well as discriminating similar mixtures. All these tasks involve discriminating odors with common ligands, implying that the associated primacy sets are correlated. This correlation can be quantified by the expected Hamming distance *d* between the primacy sets, which counts the number of glomeruli with different activities. The probability *η* that this distance is larger than 0, so that the two odor representations can be discriminated in principle, is given by

$$\begin{array}{cc} \eta (d) & \approx 1-{\left(1-\frac{d}{2N_{C}}\right)}^{N_{C}}\;; \end{array}$$ (6)

see Methods and Models. In particular, discriminating similar odors will be impossible (*η* = 0) if their primacy sets are identical (*d* = 0).

#### Discriminating uncorrelated odors

To build an intuition for this analysis, we start by considering two uncorrelated odors. In this case, each receptor type has an expected activity of 〈*a*_*n*_ 〉 = *N*_C_/*N*_R_, implying the distance d_*_ = 2*N*_C_(1 − *N*_C_ *N*_R_^−1^) and $\eta_{*}\approx 1-{\left(N_{C}/N_{R}\right)}^{N_{C}}$. Our model thus predicts that uncorrelated odors can be discriminated almost surely (*η* > 99.99% for *N*_C_ = 4 and *N*_R_ = 50). The discriminability increases strongly with *N*_C_, while the receptor repertoire size *N*_R_ has a much weaker effect in the typical case *N*_C_ ⪡ *N*_R_, similar to the scaling of the information *I* discussed above. The value η_*_ marks the upper bound for the discriminability *η*, which can be much lower for correlated odors.

#### Detecting the presence of a target odor in a background

**Fig 3:**
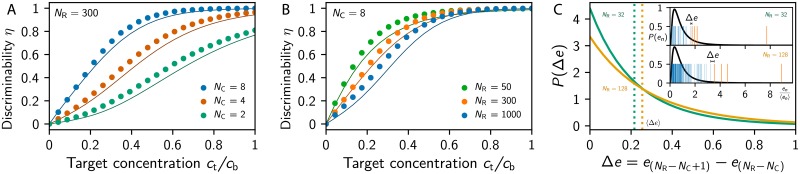
Detecting a target ligand in a background. (A-B) Probability *η* that adding a target odor at concentration *c*_t_ to a background ligand at concentration *c*_b_ can be detected using primacy coding as a function of *c*_t_/*c*_b_ for (A) various *N*_C_ at *N*_R_ = 300 and (B) various *N*_R_ at *N*_C_ = 8. Numerical simulations (dots; sample size: 10^5^) are compared to the theoretical prediction (lines) obtained using the statistical model; see Methods and Models. (C) Distribution of the difference Δ*e* between the excitations just above and below the threshold for *N*_R_ = 32 (green line) and *N*_R_ = 128 (orange line); see Methods and Models. The dotted vertical lines indicate the mean 〈Δ*e*〉, obtained from Eq (13). The inset shows *N*_R_ = 32 (upper panel) and *N*_R_ = 128 (lower panel) excitation realizations (vertical bars) drawn from the same excitation distribution (black lines). The orange bars indicate the primacy set consisting of the *N*_C_ = 4 largest excitations. (A–C) Remaining parameters are given in Fig 2A.

One simple task where odors are correlated is the detection of a target odor in a distracting background. To understand when a target can be detected, we analyze how the primacy set ***a*** changes when a single ligand at concentration *c*_t_ is added to a background ligand at concentration *c*_b_. Because of concentration-invariance, the result only depends on the relative target concentration *c*_t_/*c*_b_. Fig 3A shows that the target is easier to detect when it is more concentrated (larger *c*_t_/*c*_b_) and when more receptor types participate in the primacy code (larger *N*_C_). Conversely, the repertoire size *N*_R_ has only a weak influence, similar to the cases discussed above; see Fig 3B. Surprisingly, however, this figure also shows that dilute odors (small *c*_t_/*c*_b_) are more difficult to discriminate with larger receptor repertoires.

The fact that increasing the receptor repertoire size *N*_R_ can impede the detection of the target odor can be understood in a simplified statistical model, where we consider ensemble averages over sensitivity matrices; see Methods and Models. Since the primacy set ***a*** corresponds to the *N*_C_ receptor types with the largest excitations, ***a*** will only change when adding the target odor shuffles the excitations in the vicinity of the threshold *γ*. Intuitively, this is more likely when the associated excitation difference Δ*e* is small. Fig 3C shows that Δ*e* typically increases with *N*_R_, essentially because the distribution of the glomeruli excitation *e*_*n*_ has a heavy tail, so that sampling more excitations leads to larger gaps between the largest excitations. These larger gaps in the excitations reduce the likelihood that adding the target changes the order of the excitations and thus the primacy set. Consequently, it is more difficult to detect the target using larger repertoires. Taken together, these arguments suggest that increasing the receptor repertoire is only beneficial if the primacy dimension *Nc* is also increased.

#### Detecting the addition of a ligand to a mixture

So far, we considered simple odors consisting of single ligands. However, realistic odors are comprised of many different ligands and target odors thus also need to be detected in backgrounds of many distracting ligands. Not surprisingly, experiments in humans [45] and mice [46] have shown that targets are more difficult to identify if the background consist of many ligands. In these experiments, subjects had to indicate whether a known odor is present or not in a presented odor mixture. The probability *p*_correct_ of giving the correct answer is related to the probability *η* of obtaining enough olfactory information by $p_{\text{correct}}=\eta +\frac{1}{2}\left(1-\eta \right)$ since there is a 50% chance of choosing the correct answer even if no information is present. In the following, we compare the experimentally measured values of *p*_correct_ to the ones predicted by our model to restrict its parameters.

**Fig 4:**
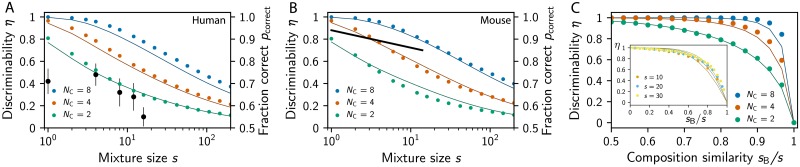
Discrimination of odor mixtures. (A,B) Probability *η* that adding a ligand to a mixture of *s* ligands changes the primacy set for various *N*_C_. The right axes display the expected fraction *p*_correct_ of correct responses in the respective go/no-go experiment. Theoretical predictions (colored symbols and lines) are compared to experimental data (black symbols and lines) for (A) humans (*N*_R_ = 300, data from [45]) and (B) mice (*N*_R_ = 1000, data from [46]). (C) Probability *η* that changing *s*_B_ ligands of a mixture of *s* ligands can be detected using primacy coding as a function of the composition similarity *s*_B_/*s* for various *N*_C_ at *N*_R_ = 300, *s* = 30, and *σ*^2^/*μ*^2^ = 0. The inset shows *η* as a function of *s*_B_/*s* for various *s* at *N*_C_ = 8, *N*_R_ = 300, and *σ*^2^/*μ*^2^ = 10. (A–C) Remaining parameters are given in Fig 2A.

For simplicity, we first ask whether the primacy set changes when a ligand is added to a background, which is necessary for discriminating the background with the target from the background without it. This analysis will provide a theoretical upper bound for the performance, allowing us to restrict model parameters. In particular, we use ensemble averages over sensitivity matrices to compute *η* and *p*_correct_ for the addition of a single ligand to a background consisting of *s* ligands, all at the same concentration. Fig 4A and 4B show that these values decrease both with larger mixture sizes *s* and smaller primacy dimension *N*_C_. Conversely, whether the receptor repertoire size of humans (*N*_R_ = 300; Fig 4A) or that of mice (*N*_R_ = 1000, Fig 4B) is considered is irrelevant for the theoretical result, while the experimental data (black symbols and lines) are significantly different. Our model suggest that the superior performance of mice could be related to a larger primacy dimension *N*_C_, although we cannot exclude the possibility that the decoding in higher regions of the brain is much more efficient in mice than in humans, e.g., because they were trained better.

A surprising finding of this analysis is that target odors can be detected more reliably when the background at a given total concentration *c*_b_ consists of many ligands. This can be seen by comparing single-ligand backgrounds (Fig 3A) with multi-ligand backgrounds (Fig 4A), where the effective target concentration is *c*_t_/*c*_b_ = 1/*s*. Considering *N*_C_ = 8, we find *η* ≈ 50% for *c*_t_/*c*_b_ ≈ 0.2 in the single-ligand case, while the ratio can be much smaller (1/*s* ≈ 0.01) for multiple ligands. This puzzling result can again be understood in the simplified statistical model, which predicts that the variance of the excitations associated with the background odor is smaller if this odor is comprised of many ligands; see Eq (9) in Methods and Models. This smaller variance implies smaller Δ*e*, so that adding the target has a higher chance of shuffling the order of the excitations to change the primacy set. The same logic implies that the target is easier to detect when the concentrations of the background ligands vary less, which is confirmed by S1 Fig. Taken together, numerical results and the statistical model suggest that a target odor is easier to notice if the background odor contains many ligands and small concentration variations.

#### Discriminating similar mixtures

To consider the discrimination of similar odors that have common ligands, we next consider odors that each contain *s* ligands, sharing *s*_B_ of them. Such odors are uncorrelated (*d* = d_*_) when they do not share any ligands (*s*_B_ = 0) and they are identical (*d* = 0) when they share all ligands (*s*_B_ = *s*). Between these two extremes, the expected distance *d* of the primacy sets, and thus the discriminability *η*, of the two odors can be determined by a numerical ensemble average over sensitivities and by the statistical model; see Methods and Models. Fig 4C shows that both methods predict that more similar odors are harder to discriminate. However, the discriminability of odors only depends on their relative similarity (the fraction of shared ligands) and is independent of the total number of ligands in the odor, consistent with psychophysical experiments [47]. Note however, that our model predicts that basically all mixtures should be easily discriminable, in contrast to the experimental result [47]. This discrepancy might be related to the fact that our model only predicts upper bounds on the encoded information and neglects the decoding in higher regions of the brain.

#### Identifying odors in a mixtures

So far, we only discussed how well odors can be discriminated, but in reality it is often necessary to identify individual odors in mixtures. To identify ligands, a decoder must compare odor representations to stored patterns. For simplicity, we here only consider a perfect decoder, which associates each representation ***a*** with an odor without any uncertainty. This allows us to derive upper bounds for the performance of odor identification without specifying a model for the olfactory processing in the brain. In essence, we use that different odors can only be identified when the associated representations differ, implying that the size of the coding space, (NRNC), must be large enough to accommodate all odors that need to be distinguished.

**Fig 5:**
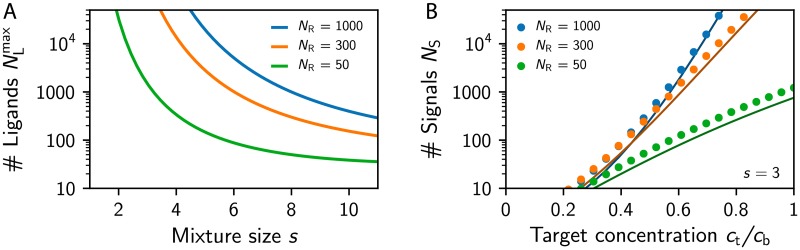
Identifying individual odors in a mixture. (A) Maximal number of ligands, $N_{L}^{\text{max}}$ such that all mixtures of *s* ligands can be discriminated using various receptor repertoire sizes *N*_R_ for *N*_C_ = 8. (B) Number *N*_S_ of ligands that could be distinguished when a ligand of concentration *c*_t_ is added to a background of *s* = 3 other ligands at concentration *c*_b_ determined from numerical (dots) and analytical (lines) ensemble averages for various *N*_R_ and *N*_C_ = 8. (A,B) Remaining parameters are given in Fig 2A.

We start by considering mixtures of *s* ligands of equal concentration and ask under what conditions all possible mixtures prepared from a library of *N*_L_ ligands are distinguishable, which is the case when (NLs)<(NRNC). Fig 5A shows that the maximal number $N_{L}^{\text{max}}$ of ligands that could possibly be identified falls off quickly with increasing mixture size *s*. This analysis implies that if humans were able to identify *N*_L_ = 1000 ligands, they could do this for mixtures of at most *s* = 6 ligands when the primacy dimension is *N*_C_ = 8. Note that this is merely an upper bound for the actual performance, since the calculation assumes that the olfactory system is optimized to identify ligands at one particular concentration, whereas natural odors contain ligands at various relative concentrations.

To see how concentration variations affect the odor identification, we next use the previously calculated mean distances *d* between odor representations to estimate how well individual ligands could be detected. In particular, the number of possible ligands that can be distinguished when they are added at concentration *c*_t_ to a mixture of *s* ligands at concentration *c*_b_ can be estimated as (NR-NCd/2), where $\frac{1}{2}d$ is the expected number of the *N*_R_ − *N*_C_ receptor types that were inactive for the background mixture and became active when the target was added. Fig 5B shows that the number of ligands that can be distinguished in this situation increases strongly with the target concentration $\frac{c_{t}}{c_{b}}$. The shown case of *s* = 3 indicates that humans would not be able to identify most ligands if their concentration was half that of the 3 background ligands. Since such concentration fluctuations are very likely to appear in natural situations and in experiments, this suggests that humans realistically can only identify ligands in mixtures of very few components, in line with experimental measurements [48, 49].

The discussion of the identification of odors is limited by the simple description of the decoder in our model. We thus derive upper bounds for the performance, assuming that a mapping of all possible odor combinations to different representations ***a*** is possible. This best-case scenario likely requires highly optimized sensitivity matrices *S*_*ni*_ and the actual performance might thus lie well below the bounds derived here. However, in realistic olfactory system, the time-course of receptor activation might provide additional information about which ligands are present. For instance, odors from multiple sources might not fully mix and thus arrive in distinguishable whiffs [50].

### Primacy coding outperforms alternative coding schemes

We showed that primacy coding contains sufficient information to perform typical olfactory tasks with experimentally measured accuracy. Although this provides some support for primacy coding, alternative encoding schemes might also be consistent with experimental data. To elucidate this, we next compare primacy coding to two alternatives, which are also based on the simple model described by Eqs (1) and (2). The first alternative is binary coding, where glomeruli become active when their excitation exceeds a constant threshold *γ* [30, 51]. The second alternative is normalized coding, where the threshold is proportional to the mean excitation, $\gamma =\alpha {N_{R}}^{-1}\sum_{n}e_{n}$, and the inhibition strength *α* determines how many glomeruli are active on average [21].

**Fig 6:**
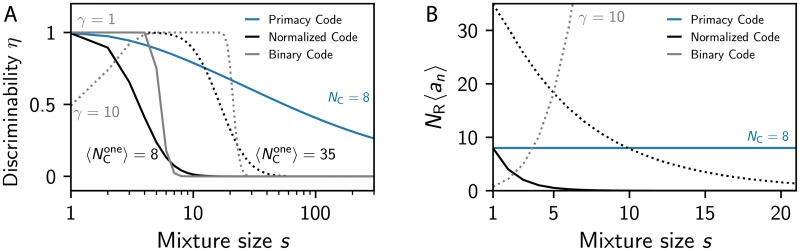
Primacy coding outperforms alternative models. Comparison of the primacy code (blue; *N*_C_ = 8) to a normalized code (black) and a binary code (gray). In the normalized code, glomeruli are active when their excitation exceeds *α* times the mean excitation [21]. Here, *α* is adjusted such that the mean number of glomeruli activated by a single ligand assumes the indicated value $\langle N_{C}^{\text{one}}\rangle$. In the binary code, glomeruli are active when their excitation exceeds the fixed threshold *γ* [30]. (A) Probability *η* that adding a ligand to a mixture of *s* ligands can be detected as a function of *s*. (B) Representation sparsity, i.e., the mean number of activated glomeruli *N*_R_〈*a_n_*〉, as a function of the mixture size *s*. (A, B) We considered fixed ligand concentrations (*σ* = 0) and the remaining parameters are given in Fig 2A.

To see how binary and normalized coding compare to primacy coding, we calculate the probability *η* that adding a ligand to a mixture of *s* ligands can be detected; see Fig 6A. The binary code strongly depends on the overall concentration of the presented odor (or, equivalently, the imposed threshold *γ*). This implies that there is only a narrow region of mixture sizes *s* where the binary code allows detecting the addition of a ligand. Conversely, the normalized code is concentration-invariant and could thus in principle discriminate odors at all intensities. However, we showed in Ref. [21] that the encoded information and the discriminability still depend strongly on the mixtures size *s* in this model. Consequently, normalized codes can only discriminate mixtures of realistic sizes when the inhibition strength *α* is very low and thus many glomeruli get activated on average.

The example of the normalized code shows that it is not sufficient to study how well different coding schemes can solve olfactory tasks, but one also needs to consider how useful this code is to the downstream decoder. Without modeling the decoder in detail, we here just propose that sparser codes are preferable since they imply fewer firing neurons, which saves energy and simplifies the downstream processing. In fact, sparse coding is typical for sensory information [19, 52]. In our model, the sparsity is given by the fraction 〈*a*_*n*_〉 of activated glomeruli and Fig 6B shows this quantity as a function of the mixture size *s*. Since larger mixtures imply a higher odor intensity, this number increases quickly in binary coding and makes this code inefficient. Conversely, the number of active glomeruli decreases strongly in normalized coding [21], which explains the poor discriminatory performance for large mixtures. In contrast, primacy coding has a constant sparsity, because it is directly controlled by the primacy count *N*_C_. Taken together, primacy coding outperforms both binary coding and normalized coding essentially because the sparsity of the representation is independent of the presented odors and can thus be adjusted to be useful and efficient over the whole range of possible odors.

The three models discussed here differ in how the statistics of the odor ***c*** affect the statistics of the output ***a***. In the binary model, the odor intensity given by the mean concentration *c*_tot_ affects the mean excitation 〈*e*_*n*_〉 and therefore the sparsity 〈*a*_*n*_〉. This clearly prevents the response from being useful over a wide concentration range. This dependence on the odor intensity is removed in normalized coding, but the variance of the excitations *e*_*n*_ still depends on the odor statistics, e.g. larger mixtures imply smaller variations in *e*_*n*_. This is problematic since it implies the excitations of fewer glomeruli exceed the fixed threshold in normalized coding, so the sparsity 〈*a*_*n*_〉 and the usefulness decrease [21]. In primacy coding, however, the mean activity $\langle a_{n}\rangle =\frac{N_{C}}{N_{R}}$ is independent of the odor statistics, so the system is useful in all situations. In fact, primacy coding can be interpreted as normalized coding with an inhibition strength *α* that depends on the non-dimensional width of the concentration distribution; see Methods and Models. Primacy coding is thus an example for global inhibition with instantaneous adaptation, which displays better performance than a simple fixed threshold *γ*. Taken together, this simple model comparison indicates that the mean response of the olfactory systems needs to be controlled and that simple normalization is not sufficient for this.

### Overly sensitive receptors degrade the coding efficiency

**Fig 7:**
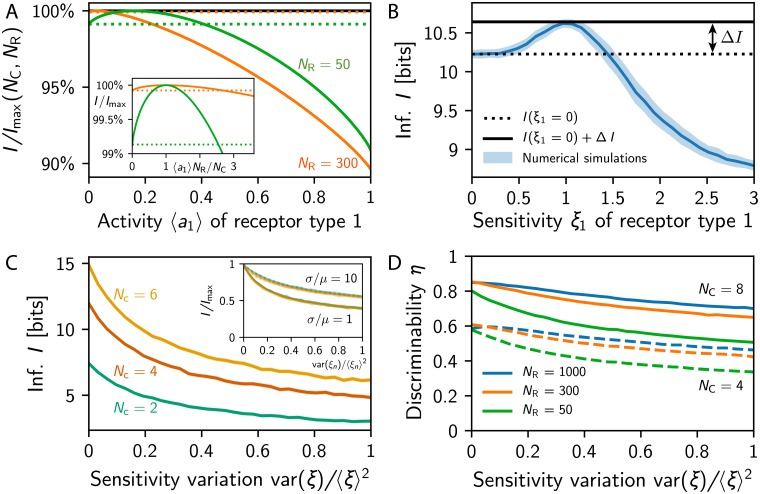
Variations in receptor activities deteriorate the array performance. (A) Information *I* relative to *I*_max_ given by Eq (5) as a function of the mean activity 〈*a*_1_〉 of the first receptor type while all others are unchanged for *N*_C_ = 8. Dotted lines indicate *I*_max_(*N*_C_, *N*_R_ − 1). Inset: Same data for the activity rescaled by *N*_C_/*N_R_*. (B) Numerical simulation of *I* as a function of the sensitivity factor *ξ*_1_ of the first receptor type for *N*_R_ = 16, *N*_C_ = 4, and *ξ*_*n*_ = 1 for *n* ≥ 2. (C) *I* for log-normally distributed sensitivity factors *ξ*_*n*_ as a function of var(*ξ*)/〈*ξ*〉^2^ for various *N*_C_ at *N*_R_ = 20. The inset shows that the scaled information *I*/*I*_max_ collapses as a function of *N*_R_ = 10, 20 and *N*_C_ = 2, 4, 6 for different widths *σ*/*μ* of the concentration distribution. (D) Probability *η* that adding a ligand to a mixture of *s* = 10 ligands changes the primacy set as a function of var(*ξ*)/〈*ξ*〉^2^ for various *N*_R_ and *N*_C_. (B–D) Shown are numerical simulations with *N*_L_ = 512, *μ* = *σ* = 1, and λ = 1 as well as *s* = 16 in (B,C) and *s* = 10 in (D).

So far, we calculated the transmitted information and tested the discrimination performance of primacy coding under the assumption that all receptor types behave similarly. In fact, we established that the maximal information is achieved when all receptor types are activated with equal probability *N*_C_/*N*_R_. However, neither the receptor sensitivities nor the odors themselves are distributed equally in realistic situations. Variations in these quantities affect the transmitted information and thus the usefulness of the primacy code. For instance, the transmitted information decreases if a single receptor is activated less often than all the others; see Fig 7A. This effect is small, since in the worst case the receptor is never active and the transmitted information thus corresponds to an array with this receptor removed. Conversely, having a receptor that is active more often than all others can have a much more severe effect; see Fig 7A. In fact, if the receptor type is more than three times as active, the transmitted information *I* is lower than if the receptor type was remove completely; see Methods and Models. This indicates that receptors can shadow the response of other receptors and thus be detrimental to the overall array when they are overly sensitive.

The effect of varying receptor sensitivities can be studied in our model of primacy coding by discussing more general sensitivities matrices. We consider $S_{ni}=\xi_{n}S_{ni}^{\text{iid}}$, where each receptor type can have a different sensitivity factor *ξ*_*n*_, which modulates the uniform sensitivity matrix $S_{ni}^{\text{iid}}$ where each entry is independently chosen from the same log-normal distribution. The case of homogeneous sensitivities that we discusses so far thus corresponds to *ξ*_*n*_ = 1 for *n* = 1, 2, …, N_R_.

To investigate the effect of heterogeneous sensitivities, we start by varying the sensitivity factor of one receptor type while keeping all others untouched, i.e., we change *ξ*_1_ while keeping *ξ*_*n*_ = 1 for *n* ≥ 2. There are three simple limits that we can discuss immediately. For *ξ*_1_ = 0, the first receptor type will never become active, the array behaves as if this type was not present, and the transmitted information is approximately *I*_max_(*N*_C_, *N*_R_ − 1). This value is lower than the maximally transmitted information *I*_max_(*N*_C_, *N*_R_) reached for the symmetric case *ξ*_1_ = 1. However, the associated information loss Δ*I* = *I*_max_(*N*_C_, *N*_R_) − *I*_max_(*N*_C_, *N*_R_ − 1) ≈ *N*_C_/(*N*_R_ ln 2) is relatively small in large receptor arrays (*N*_R_ ≫ *N*_C_); see Fig 7B. Conversely, the transmitted information can be affected much more severely if the sensitivity of the first receptor type is increased beyond *ξ*_1_ = 1 and the receptors will thus be active more often than the others. In the extreme case of *ξ*_1_ → ∞, the first receptor type will always be active and thus not contribute any information. Since this receptor type would always be part of the primacy set, the information transmitted by the remaining receptor types is approximately *I*_max_(*N*_C_ − 1, *N*_R_ − 1), which is smaller than *I*_max_(*N*_C_, *N*_R_ − 1) in the typical case *N*_R_ ≫ *N*_C_. Consequently, an overly active receptor type can be worse than not having this type at all under primacy coding.

The fact that overly sensitive receptors are detrimental to the transmitted information is also visible in numerical simulations. Fig 7B shows ensemble averages of the information *I* transmitted by receptor arrays as a function of the sensitivity factor *ξ*_1_. As qualitatively argued above, *I* is maximal for *ξ*_1_ = 1 and it is slightly lower for smaller *ξ*_1_ since the receptor type is active less often. In contrast, for *ξ*_1_ > 1, *I* decreases dramatically and falls below the value of *ξ*_1_ = 0 for *ξ*_1_ ≳ 1.5. These data suggest that it would be better to remove receptor types that exhibit a 50% higher sensitivity than the other types.

To see whether overly sensitive receptor types are also detrimental when all types have varying sensitivities, we next considering sensitivity factors *ξ*_*n*_ distributed according to a lognormal distribution. Numerical results shown in Fig 7C indicate that the transmitted information indeed decreases with increasing variance var(*ξ*_*n*_) of the sensitivity factors. In fact, a variation of var(*ξ*_*n*_)/〈*ξ*_*n*_〉^2^ = 0.5 already implies a reduction of the transmitted information by almost 50% for small concentration variations *σ*/*μ* = 1. If the odor concentrations vary more, the information degradation is less severe, but the same trend is visible. Interestingly, rescaling the information by the maximal information *I*_max_ given in Eq (5) collapses the curves for all dimensions *N*_C_ and *N*_R_, suggesting that this analysis also holds for realistic receptor repertoire sizes. Note that the reduced transmitted information also implies poorer odor discrimination performance; see Fig 7D. Taken together, this provides a strong selective pressure to limit the variability of the receptor sensitivities so overly sensitive receptors do not dominate the whole array.

## Discussion

We analyzed a simple model of neural representations of olfactory stimuli, where odors are identified by the *N*_C_ strongest responding receptor types. This version of primacy coding provides a sparse representation of the odor identity, which is independent of the odor intensity. We showed using numerical simulations and a statistical model that the primacy dimension *N*_C_ strongly affects the transmitted information and the discriminability of odors. Interestingly, already for small values of *N*_C_ ≲ 10, the typical olfactory discrimination tasks can be carried out with performances close to experimentally measured ones. Conversely, the number *N*_R_ of receptor types does not strongly affect the coding capacity and the discriminability of similar odors, in accordance with lesion experiments. Our model even indicates that lowering *N*_R_ can improve the identification of a target ligand in a background.

The advantage of our simple model is that we can analyze its behavior in depth and explicitly link the statistical properties of the olfactory system to data from psycho-physical experiments. In particular, we predict how likely two different odors drawn from a particular statistics can be distinguished. For instance, our model implies that target odors are easier to detect if disturbing backgrounds consist of many ligands. We generally find that representations are sensitive to the relative concentration of ligands in mixtures and that dilute components are basically completely shadowed. Conversely, for fixed ligand concentrations mixtures can typically be discriminated very well. However, identifying the individual ligands in mixture is only possible for mixtures with few components. In any case, our results suggest that the primacy code formed in the olfactory bulb is more useful to identify odors in the subsequent olfactory cortex than simple alternatives, essentially because the statistics of the representations are independent of the odor statistics.

Our model predicts that receptors are only useful if their likelihood to respond to incoming odors is similar. This is because receptor types that are overly sensitive and respond strongly to many odors could dominate other types and thus degrade the total information. In fact, having a receptor type that is 50% more sensitive than others, and thus responds about three times as often, can lead to less transmitted information than when this type is absent. This observation is related to the primacy hull discussed in [38], which also predicts strong restrictions on the receptor sensitivities stemming from primacy coding. Various strategies could play a role in keeping the activity of the receptor types similar [53]: On timescales as short as a single sniff, the inhibition strength could be adjusted to regulate the relative importance of receptor excitations [54]. On longer timescales of several weeks, there are changes of the receptor copy number that directly affect the sensitivity of the glomeruli [55–57] and the processing neurons in the olfactory bulb [58, 59]. Receptor copy number adaptations influence the signal-to-noise ratio at the receptor level, so the copy number could be increased to improve the detection of frequently appearing odors [60]. In contrast, we predict a decrease of the copy number of overly sensitive receptor types that respond often. Combining the two alternatives, receptor copy numbers could be controlled such that noise is suppressed sufficiently while ensuring that single receptor types do not dominate the array. Finally, receptor sensitivities can also be adjusted by genetic modifications on evolutionary timescales [61, 62]. Moreover, direct feedback from higher regions of the brain could modify the processing of olfactory signals, e.g., in response to the behavioral state [7]. Although our work shows that the activities of the receptors need to be balanced, the actual distribution of the sensitivities matters much less. For instance, log-uniform distributions, which have been suggested to describe realistic receptor arrays [51, 63], lead to similar odor discriminability as log-normally distributed sensitivities; see S2 Fig.

Our results raise the question why mice have 20 times as many receptor types than flies although the transmitted information under primacy coding is only increased by a factor of 2 (see Eq (5)) and the odor discriminability is hardly affected by the receptor repertoire size (see Fig 4). The apparent usefulness of large receptor repertoires hints at roles of the olfactory system beyond transmitting the maximal information and discriminating average odors. For instance, having many receptor types might help to hardwire innate olfactory behavior when receptors are narrowly tuned to odors. In this case, our model would only apply to the fraction of the receptor types that are broadly tuned and are not connected to innate behavior. Alternatively, having many receptor types might be advantageous to discriminate very similar odor mixtures, to cover a larger dynamic range in concentrations of individual ligands, or to allow for a larger variation in average sensitivities, enabling quick adaptation to new environments. Finally, biophysical constraints of the receptor structure might imply that many receptors are required to cover a large part of chemical space.

We discussed the simplest version of primacy coding with a minimal receptor model and a constant primacy dimension *N*_C_ implemented by a hard threshold. This model neglects the complex interactions of ligands at the olfactory receptors, which can affect perception [64]. In particular, antagonistic effects can already provide some normalization at the level of receptors [65]. Generally, it is likely that many mechanisms contribute to the overall normalization of the receptor response [66]. A more realistic model of primacy coding might also consider a softer threshold, where receptor types with larger excitation are given higher weight in the downstream interpretation, which is related to rank coding [22]. In this case, information from fewer glomeruli might be sufficient to identify odors, since the rank carries additional information. Realistic olfactory systems could also use a timing code, taking into account more and more receptor types (with decreasing excitation) until an odor is identified confidently. Such a system could explain that the response dynamics in experiments depend on the task [67, 68]. Generally, a better understanding of the temporal structure of the olfactory code [8, 69–73] might allow to derive more detailed models. These could rely on attractor dynamics that are guided by the excitations and thus respond stronger to the early and large excitations [74, 75].

## Methods and models

### Numerical simulations

All numerical simulations are based on ensemble averages over sensitivity matrices *S*_*ni*_. The elements of *S*_*ni*_ are drawn independently from a log-normal distribution with $\mathrm{var}\left(S_{ni}\right)/{\bar{S}}^{2}=1.72$ corresponding to λ = 1. In Figs 2A and 7B–7D, an additional ensemble average over odors ***c*** is performed using the distribution *P*_env_(***c***). Here, odors ***c*** are chosen by first determining which of the *N*_L_ ligands are present using a Bernoulli distribution with probability *p* = *s*/*N*_L_ and then independently drawing their concentration from a log-normal distribution with mean *μ* and standard deviation *σ*. In all simulations the primacy set ***a*** corresponding to ***c*** is given by the *N*_C_ receptors with the highest excitation calculated from Eq (1). Statistics of ***a*** and the transmitted information *I* given by Eq (4) are determined by repeating this procedure 10^5^ and 10^7^ times, respectively.

### Statistical model

In order to obtain deeper insights into the numerical results, we also develop analytical approximations using a statistical description of all involved quantities, which is based on accounting for the means and variances of the respective distributions. For instance, the statistics of the output ***a*** given by Eqs (1)–(3) can be estimated using ensemble averages of sensitivity matrices for different odors ***c***, similar to our treatment presented in [21] and [30]. In particular, Eq (1) implies that the effects of different ligands are additive. Since the log-normal distribution describing the sensitivities is narrow (λ = 1), the excitations *e*_*n*_ are also well approximated by a log-normal distribution with mean ${\langle e_{n}\rangle }_{S}=\bar{S}\sum_{i}c_{i}$ and variance and ${\mathrm{var}}_{S}\left(e_{n}\right)=\mathrm{var}\left(S_{ni}\right)\sum_{i}c_{i}^{2}$ [76], whereas correlations are negligible [21]. The probability that the excitation *e*_*n*_ exceeds the threshold *γ*, and the associated receptor type is thus part of the primacy set, reads

$$\begin{array}{c} {\left\langle a_{n}\right\rangle }_{S}=1-G(\frac{\gamma (c)}{{\left\langle e_{n}\right\rangle }_{S}};\zeta \left(c\right)) \end{array}$$ (7)

with

$$\begin{array}{c} G\left(x;\zeta \right)=\frac{1}{2}+\frac{1}{2}\mathrm{erf}(\frac{\zeta +\log(x)}{2\zeta^{\frac{1}{2}}}) \end{array}$$ (8)

being the cumulative density function of a log-normal distribution with 〈*x*〉 = 1 and var(*x*) = exp(2*ζ*) − 1. The width of the distribution is determined by the positive parameter $\zeta =\frac{1}{2}\ln\left(1+\mathrm{var}\left(e_{n}\right)/{\langle e_{n}\rangle }^{2}\right)$, which reads

$$\begin{array}{c} \zeta \left(c\right)=\frac{1}{2}\ln[1+(e^{\lambda^{2}}-1)\frac{\sum_{i}c_{i}^{2}}{{\left(\sum_{i}c_{i}\right)}^{2}}] \end{array}$$ (9)

for an ensemble average over sensitivities. Note that *ζ* is concentration-invariant, since it does not change when the concentration vector ***c*** is multiplied by a constant factor. In the simple case of ligands that are distributed according to *P*_env_(***c***), we find ${\langle \left(\sum_{i}c_{i}^{2}\right){\left(\sum_{i}c_{i}\right)}^{-2}\rangle }_{c}=s^{-1}\left(1+\sigma^{2}/\mu^{2}\right)$. Consequently, the distribution width *ζ* is large for broadly distributed sensitivities (large λ), few ligands in an odor (small *s*), and wide concentration distributions (large *σ*/*μ*).

The constraint Eq (3) implies 〈*a_n_*〉 = *N*_C_/*N*_R_, so that the mean threshold reads

$$\begin{array}{c} \left\langle \gamma \right\rangle ={\left\langle e_{n}\right\rangle }_{S}\cdot G^{-1}(1-\frac{N_{C}}{N_{R}};\zeta )\;, \end{array}$$ (10)

where *G*^−1^ is the inverse function of *G* defined in Eq (8). Using this expression as an estimate for *γ* in Eq (7) results in concentration-invariant activities *a*_*n*_, since 〈*γ*〉 is proportional to the excitation 〈*e*_*n*_〉. This situation is comparable to simple normalized representations resulting from the threshold *γ* = *α*〈*e*_*n*_〉, where *α* is a constant inhibition strength [21]. In fact, primacy coding can be interpreted as global inhibition with an inhibition threshold depending on the width of the excitation distribution, $\alpha =G^{-1}\left(1-N_{C}{N_{R}}^{-1};\zeta \right)$.

#### Inter-excitation intervals

The expected difference between excitations corresponding to a given odor ***c*** can be studied using order statistics, where excitations are re-indexed such that they are ordered, $e_{\left(1\right)}<e_{\left(2\right)}<\ldots <e_{\left(N_{R}\right)}$. For simplicity, we consider the case where the excitations *e*_*n*_ are distributed identically when considering all odors according to *P*_env_(***c***). Denoting the cumulative distribution function of the excitations by $F\left(e\right)=G\left(\frac{e}{\langle e_{n}\rangle };\zeta \right)$ and the associated probability density function by *f*(*e*), the probability density function associated with the excitation *e*_(*n*)_ at rank *n* reads [77]

$$\begin{array}{c} f_{E_{\left(n\right)}}\left(e\right)=\frac{N_{R}!\;f\left(e\right)}{\left(n-1\right)!\;\left(N_{R}-n\right)!}\;F^{n-1}\left(e\right)\;{\left[1-F\left(e\right)\right]}^{N_{R}-n}\;. \end{array}$$ (11)

The joint distribution of *E*_(*n*)_ and *E*_(*m*)_, 1 ≤ *n* < *m* ≤ *N*_R_, reads [77]

$$\begin{array}{c} f_{E_{\left(n\right)},E_{\left(m\right)}}\left(e_{n},e_{m}\right)=\frac{N_{R}!\;f\left(e_{n}\right)f\left(e_{m}\right)}{\left(n-1\right)!\left(m-n-1\right)!\left(N_{R}-m\right)!}\\ \cdot F^{n-1}\left(e_{n}\right){\left[1-F\left(e_{m}\right)\right]}^{N_{R}-m}\;{\left[F\left(e_{m}\right)-F\left(e_{n}\right)\right]}^{m-n-1}\;. \end{array}$$ (12)

Consequently, the distribution of the difference Δ*e* = *e*_(*n*)_ − *e*_(*n*−1)_ of consecutive excitations is

$$\begin{array}{c} f_{\Delta E}\left(\Delta e;n\right)=\int_{0}^{\infty }f_{E_{\left(n-1\right)},E_{\left(n\right)}}\left(y,\Delta e+y\right)\;\text{d}y\;. \end{array}$$ (13)

Hence, the expected difference 〈Δ*e*〉 = ∫ *xf*_Δ*E*_(*x*; *N*_R_ − *N*_C_ − 1) d*x* between the strongest excited inactive receptor type and the weakest active receptor type can be evaluated.

#### Discriminability of primacy set

The expected number *d* of changes in the primacy set ***a*** when a target odor ***c***^t^ is added to some background ***c***^b^ reads

$$\begin{array}{c} d=N_{R}\cdot \left(p_{\text{on}}+p_{\text{off}}\right)\;, \end{array}$$ (14)

where *p*_on_ is the probability that a receptor type that was inactive for ***c***^b^ is turned on by the perturbation ***c***^t^ and *p*_off_ is the probability that a receptor type that was active is turned off. Both probabilities depend on the excitation thresholds *γ*^(1)^ and *γ*^(2)^ associated with the odors ***c***^b^ and ***c***^b^ + ***c***^t^, respectively, which can be estimated from Eq (10) using the respective excitation statistics. With this, *p*_on_ follows from the probability that the excitation was at the value *x* below *γ*^(1)^ and the additional excitation by the target brings the total excitation above *γ*^(2)^,

$$\begin{array}{c} p_{\text{on}}\approx \int_{0}^{\gamma^{\left(1\right)}}[1-G(\frac{\gamma^{\left(2\right)}-x}{{\left\langle e_{n}^{t}\right\rangle }_{S}};\zeta^{t})]g(\frac{x}{{\left\langle e_{n}^{b}\right\rangle }_{S}};\zeta^{b})\;\text{d}x\;, \end{array}$$ (15)

where *g*(*e*; *ζ*) is the probability density function associated with *G*(*e*; *ζ*) given in Eq (8). Here, ${\langle e_{n}^{j}\rangle }_{S}$ and *ζ*^*j*^ describe the excitation statistics of the target (*j* = t) and the background (*j* = b). Similarly, we obtain

$$\begin{array}{c} p_{\text{off}}\approx \int_{\gamma^{\left(1\right)}}^{\gamma^{\left(2\right)}}\;\;\;G(\frac{\gamma^{\left(2\right)}-x}{{\left\langle e_{n}^{t}\right\rangle }_{S}};\zeta^{t})\;g(\frac{x}{{\left\langle e_{n}^{b}\right\rangle }_{S}};\zeta^{b})\;\text{d}x\;, \end{array}$$ (16)

so we can use Eq (14) to calculate the expected Hamming distance *d*. Note that *γ*^(1)^ and *γ*^(2)^ depend on *N*_R_, so the distance *d* does not scale trivially with *N*_R_, in contrast to the case of normalized representations [21].

We use Eqs (14)–(16) to calculate *d* when a target ligand with concentration *c*_t_ is added to a background ligand at concentration *c*_b_. The associated statistics of the excitations obey

$$\begin{array}{cc} {\left\langle e_{n}^{t}\right\rangle }_{S}=\frac{c_{t}}{c_{b}}{\left\langle e_{n}^{b}\right\rangle }_{S} & {\mathrm{var}}_{S}\left(e_{n}^{t}\right)={\left(\frac{c_{t}}{c_{b}}\right)}^{2}{\mathrm{var}}_{S}\left(e_{n}^{b}\right) \end{array}$$ (17)

and ${\mathrm{var}}_{S}\left(e_{n}^{b}\right)/{\langle e_{n}^{b}\rangle }_{S}^{2}$ follows from chosen values of *σ*/*μ* and λ. Similarly, when a ligand with concentration *c* is added to a mixture of *s* ligands, all at concentration *c*, we have

$$\begin{array}{ccc} {\left\langle e_{n}^{t}\right\rangle }_{S} & =s^{-1}{\left\langle e_{n}^{b}\right\rangle }_{S} & {\mathrm{var}}_{S}\left(e_{n}^{t}\right)=s^{-1}{\mathrm{var}}_{S}\left(e_{n}^{b}\right)\;. \end{array}$$ (18)

The third case of correlated odors that we discuss in the main text concerns two odor mixtures of equal size *s* sharing *s*_B_ of the ligands. In this case, the excitation threshold *γ* is the same for both odors and we can express the probability *p*_xor_ that a receptor type is excited by one mixture but not the other as

$$\begin{array}{c} p_{\text{xor}}=\int_{0}^{\gamma }G(\frac{\gamma -x}{{\left\langle e_{n}^{D}\right\rangle }_{S}};\zeta^{D})[1-G(\frac{\gamma -x}{{\left\langle e_{n}^{D}\right\rangle }_{S}};\zeta^{D})]g(\frac{x}{{\left\langle e_{n}^{B}\right\rangle }_{S}};\zeta^{B})\text{d}x\;, \end{array}$$ (19)

where the parameters ${\langle e_{n}^{j}\rangle }_{S}$ and *ζ*^*j*^ need to be evaluated for the excitations associated with the *s*_B_ ligands that are the same (*j* = B) and the *s* − *s*_B_ ligands that are different (*j* = D) between the two mixtures. Taken together, the expected distance reads *d* = 2*N*_R_*p*_xor_ and we recover *d* = d_*_ for unrelated mixtures (*s*_B_ = 0) and *d* = 0 for identical mixtures (*s*_B_ = *s*).

The calculated distances *d* between activities can be used to estimate the probability *η* that the two involved odors can be discriminated. Assuming that glomeruli are independent, the distribution of distances between two activities can be modeled as a binomial distribution over the possible values {0, 2, 4, …, 2*N*_C_} with a mean equal to *d*. The probability *η* that the representations differ in at least one glomerulus, i.e. that the distance is larger than 0, then reads

$$\begin{array}{cc} \eta & \approx 1-{\left(1-\frac{d}{2N_{C}}\right)}^{N_{C}}\;, \end{array}$$ (20)

which reduces to *η* ≈ 1 − *e*^−*d*/2^ in the limit *N*_C_ ≫ 1.

#### Information transmitted by diverse receptors

In the case where the primacy sets ***a*** can be partitioned into *N*_*M*_ groups with all elements within a group appearing with the same probability, we can write the information *I* given by Eq (4) as

$$\begin{array}{c} I=-\sum_{m=1}^{N_{M}}p_{m}\log_{2}(\frac{p_{m}}{M_{m}})\;, \end{array}$$ (21)

where *M*_*m*_ is the number of elements within group *m* and *p*_*m*_ is the probability that group *m* appears in the output, such that ∑mMm=(NRNC) and ∑_*m*_ *p*_*m*_ = 1. In the simple case of one receptor type with deviating statistics, we have *N*_*M*_ = 2 with

$$\begin{array}{cc} p_{1}=\langle a_{1}\rangle & p_{2}=1-\langle a_{1}\rangle \end{array}$$ (22a)

M1=(NR-1NC-1)M2=(NR-1NC) (22b)

while the remaining activities are 〈*a_n_*〉 = (*N*_C_ − 〈*a*_1_〉)/(*N*_R_ − 1) for *n* ≥ 2 to obey Eq (3). For *p*_1_ = 0, Eq (21) reduces to *I* = *I*_max_(*N*_C_, *N*_R_ − 1), whereas the maximum *I* = *I*_max_(*N*_C_, *N*_R_) is reached for *p*_1_ = *N*_C_/*N*_R_. The information decreases for larger *p*_1_ and eventually reaches values lower than *I*_max_(*N*_C_, *N*_R_ − 1) when $p_{1}=p_{1}^{\text{max}}$. For $p_{1}>p_{1}^{\text{max}}$, it would thus be advantageous to remove this receptor type. Using (nk)≈nk/k! and expanding Eq (21) around *p*_1_ = *eN*_R_/(*N*_R_ − 1), we find

$$\begin{array}{c} p_{1}^{\text{max}}\approx \frac{1}{\log(1-\frac{eN_{C}}{N_{R}-1})-1}+1=\frac{eN_{C}}{N_{R}}+O({\left[\frac{N_{C}}{N_{R}}\right]}^{2}) \end{array}$$ (23)

in the limit *N*_R_ ≫ *N*_C_ of large repertoires, so $p_{1}^{\text{max}}\approx e\;N_{C}/N_{R}$.

## Supporting information

S1 Fig Discrimination of odor mixtures with varying ligand concentrations. Probability *η* that adding a ligand to a mixture of *s* ligands changes the primacy set for various *N*_C_. (A) *η* as a function of *s* for various widths *σ*^2^/*μ*^2^ of the ligand concentration distribution at *N*_R_ = 300 and *N*_C_ = 8. (B,C) *η* as a function of *s* for various *N*_C_ at *σ*^2^/*μ*^2^ = 10. Ensemble averages of the model (colored symbols) are compared to experimental data (black symbols and lines) for (B) humans (*N*_R_ = 300, data from [45]) and (C) mice (*N*_R_ = 1000, data from [46]). The right axes display the expected fraction of correct responses in the respective go/no-go experiment. (A–C) Remaining parameters are given in Fig 2A.
(EPS)

S2 FigLog-uniform distributed sensitivities behave similar to log-normal distributed ones under primacy coding.(A–B) Probability *η* that adding a ligand at concentration *c*_t_ to a background ligand at concentration *c*_b_ changes the primacy set ***a*** as a function of *c*_t_/*c*_b_ for (A) various *N*_C_ at *N*_R_ = 300 and (B) various *N*_R_ at *N*_C_ = 8. (C) Probability *η* that adding a ligand to a mixture of *s* ligands changes the primacy set as a function of *s* for various *N*_C_ and *N*_R_ = 300. (A–C) Shown are numerical simulations (dots; sample size: 10^5^) for *N*_L_ = 512, *σ*/*μ* = 0, and $\mathrm{var}\left(S_{ni}\right)/{\bar{S}}^{2}=7$, so the log-uniform distributed sensitivities span about 7 orders of magnitude. Note that the three panels are similar to Figs 3A, 3B and 4A, respectively, implying that log-uniform and log-normal distributed *S*_*ni*_ behave similarly.(EPS)
